# Supplementary material for: A comparison of new cardiovascular endurance test using the 2-minute marching test vs. 6-minute walk test in healthy volunteers: A crossover randomized controlled trial
Source: PLoS One. 2024 Aug 28;19(8):e0307650. doi: 10.1371/journal.pone.0307650 (PMC11356390; doi:10.1371/journal.pone.0307650)
Supplement: S1 File — (PDF) [file pone.0307650.s001.pdf]

CONSORT checklist of information to include when reporting randomised crossover trials			
**Section/topic**	**Item No**	**Description**	**Page No***
Title†	1a	Identification as a randomised crossover trial in the title	1
Abstract†	1b	Specify a crossover design and report all information outlined in Table 2 of Dwan et al, *BMJ* 2019;366:l4378; doi: 10.1136/bmj.l4378	2
Introduction:			
Background‡	2a	Scientific background and explanation of rationale	3-5
Objectives‡	2b	Specific objectives or hypotheses	5
Methods:			
Trial design†	3a	Rationale for a crossover design. Description of the design features including allocation ratio, especially the number and duration of periods, duration of washout period, and consideration of carry over effect	5
Change from protocol‡	3b	Important changes to methods after trial commencement (such as eligibility criteria), with reasons	None
Participants‡	4a	Eligibility criteria for participants	7
Settings and location‡	4b	Settings and locations where the data were collected	7-8
Interventions†	5	The interventions with sufficient details to allow replication, including how and when they were actually administered	None
Outcomes‡	6a	Completely defined prespecified primary and secondary outcome measures, including how and when they were assessed	7-8
Changes to outcomes‡	6b	Any changes to trial outcomes after the trial commenced, with reasons	None
Sample size†	7a	How sample size was determined, accounting for within participant variability	7
Interim analyses and stopping guidelines‡	7b	When applicable, explanation of any interim analyses and stopping guidelines	None
Randomisation:			
Sequence generation‡	8a	Method used to generate the random allocation sequence	5-6
Sequence generation‡	8b	Type of randomisation; details of any restriction (such as blocking and block size)	5-6
Allocation concealment‡	9	Mechanism used to implement the random allocation sequence§ (such as sequentially numbered mechanism containers), describing any steps taken to conceal the sequence until interventions were assigned	5-6
Implementation†	10	Who generated the random allocation sequence,§ who enrolled participants, and who assigned participants to the sequence of interventions	8-9
Blinding‡	11a	If done, who was blinded after assignment to interventions (for example, participants, care providers, those assessing outcomes) and how	8-9
Similarity of interventions‡	11b	If relevant, description of the similarity of interventions	None
Statistical methods†	12a	Statistical methods used to compare groups for primary and secondary outcomes which are appropriate for crossover design (that is, based on within participant comparison)	9
Additional analyses‡	12b	Methods for additional analyses, such as subgroup analyses and adjusted analyses	None
Results			
Participant flow (a diagram is recommended)†	13a	The numbers of participants who were randomly assigned, received intended treatment, and were analysed stronglyfor the primary outcome, separately for each sequence and period	Figure 1; p.5
Losses and exclusions†	13b	No of participants excluded at each stage, with reasons, separately for each sequence and period	Figure 1; p.5
Recruitment‡	14a	Dates defining the periods of recruitment and follow-up	6
Trial end‡	14b	Why the trial ended or was stopped	6
Baseline data†	15	A table showing baseline demographic and clinical characteristics by sequence and period	10
Numbers analysed†	16	Number of participants (denominator) included in each analysis and whether the analysis was by original assigned groups	10
Outcomes and estimation†	17a	For each primary and secondary outcome, results including estimated effect size and its precision (such as 95% confidence interval) should be based on within participant comparisons.¶ In addition, results for each intervention in each period are recommended	11-18
Binary outcomes‡	17b	For binary outcomes, presentation of both absolute and relative effect sizes is recommended	None
Ancillary analyses‡	18	Results of any other analyses performed, including subgroup analyses and adjusted analyses, distinguishing prespecified from exploratory	15-16
Harms†	19	Describe all important harms or untended effects in a way that accounts for the design (for specific guidance, see CONSORT for harms¥)	21
Discussion:			

BMJ: first published as 10.1136/bmj.l4378 on 31 July 2019. Downloaded from http://www.bmj.com on 10 October 2019 at University of Bristol Library. Protected by copyright.

Limitations†	20	Trial limitations, addressing sources of potential bias, imprecision, and if relevant, multiplicity of analyses.<br>Consider potential carry over effects	23
Generalisability‡	21	Generalisability (external validity, applicability) of the trial findings	19-20
Interpretation‡	22	Interpretation consistent with results, balancing benefits and harms, and considering other relevant evidence	18-23
Other information:			
Registration‡	23	Registration number and name of trial registry	6
Protocol‡	24	Where the full trial protocol can be accessed, if available	Figure 3; p.6
Funding‡	25	Sources of funding and other support (such as supply of drugs), role of funders	None

CONSORT=Consolidated Standards of Reporting Trials.
*Note: page numbers are optional depending on journal requirements.
†Modified original CONSORT item.
‡Unmodified CONSORT item.
§Random sequence here refers to a list of random orders, typically generated through a computer program. This should not be confused with the sequence of interventions in a randomised crossover trial, for example receiving intervention A before B for an individual trial participant.
¶A within participant comparison takes into account the correlation between measurements for each participant because they act as their own control, therefore measurements are not independent.
¥ Ioannidis JP, Evans SJ, Gotzsche PC, et al. CONSORT Group.Better reporting of harms in randomized trials: an extension of the CONSORT statement. *Ann Intern Med* 2004;141:781-8. doi:10.7326/0003-4819-141-10-200411160-00009
