# Supplementary material for: A comparison of new cardiovascular endurance test using the 2-minute marching test vs. 6-minute walk test in healthy volunteers: A crossover randomized controlled trial
Source: PLoS One. 2024 Aug 28;19(8):e0307650. doi: 10.1371/journal.pone.0307650 (PMC11356390; doi:10.1371/journal.pone.0307650)
Supplement: S2 File — (PDF) [file pone.0307650.s002.pdf]

Document No.1

BHQ-IRB Submission Form

Protocol Summary

Rationale	This study investigates a new cardiovascular endurance assessment. The results will be used to evaluate cardiovascular endurance during exercise and develop oxygen desaturation indicators in patients with lung disease, cardiovascular disease, COVID-19, and Post COVID-19 patients who cannot attend hospital follow-up assessments.
Objectives	To compare the effects of the cardiovascular endurance test (VO2max) between the 2-minute marching test (2-MMT) and the 6-minute walk test (6-MWT).
Study design	A crossover randomized controlled trial
Sample size	254 participants
Study schedule and procedures)	1. The researcher presented the proposal to the Human Research Ethics Committee of Bangkok Hospital Headquarters.<br>2. The researcher collected data in accordance with the study protocol and IRB approval.<br>3. Analyzed data according to objectives and summarized research results.
Expected results and benefits	1) Participants benefit: Participants will know about their cardiovascular system using the new cardiovascular test 2-MMTand 6-MWT.<br>2) Career benefits: This study will serve as a model for future

	cardiovascular endurance disease research, extending the findings to different medical fields.<br>3) Social benefits: educational institutions/academic institutions can cite and conduct additional research.

Protocol Identification and Investigator

1. **Title**: Comparison of the New Cardiovascular Endurance test using the 2-Minute Marching Test vs. 6-Minute Walk Test in Healthy Volunteers: A Crossover Randomized Controlled Trial

2. **Principle Investigator,** Sucheela Jisarojito, MD.

❑**Position:** Director of Rehabilitation Center, Bangkok Hospital Headquarters

❑ **Department:** Rehabilitation Center, Bangkok Hospital Headquarters

❑ **Educational Qualification**: Board of Physical Medicine and Rehabilitation (PMR)

Contact: Rehabilitation Center, Bangkok Hospital Headquarters / 02-3103139

Mobile phone: 085-8119338

E-mail address: sucheela.ji@bangkokhospital.com

2.1 Research workload and responsibilities

The researcher has several research projects under the supervision of ....-..........projects. It is estimated that the total number of research participants/volunteers who are in the project and follow-up is.......-.... person

Human ethics experience

❑ The researcher has received training in human ethics. Year..

☑ The investigator has completed the Good Clinical Research Training (GCP) 2020 (Certificate as attached).

❑ The researcher is untrained. However, the research team's potential to meet international standards will be developed as follows.

2.2 The stakeholder's funding sources/research drugs/research tools.

stakeholder	Principle Investigator
Do you or your family hold >5% shares, work with the company, or earn >300,000 baht/year from funding before the research. (Bangkok Hospital Headquarters and Bangkok Hospital Group salaries excluded)	❑ Yes<br>☑ No
Are you in a management or academic position at a company/research funding agency? (Bangkok Hospital Headquarters and the Bangkok Hospital Group excluded)	❑ Yes<br>☑ No
Are you an academic, financial, legal advisor, or regular speaker on funding products	❑ Yes<br>☑ No
Are you involved in the production, purchase, sale, lease, registration, or supply of research-related products?	❑ Yes<br>☑ No
Have you received domestic and international conference funding in the previous year or earned speaker fees >300,000 baht? (Bangkok Hospital Headquarters and Bangkok Hospital Group salaries excluded)	❑ Yes<br>☑ No

3. Co-investigators

1) Lt.Col. Suchai Surapichpong, Ph.D.

☑ Position: Consultant, Rehabilitation Center, Bangkok Hospital Headquarters

☑ Department: Rehabilitation Center, Bangkok Hospital Headquarters

☑ Education: Ph.D.

Contact: Rehabilitation Center, Bangkok Hospital Headquarters / 02-3103139

Mobile Phone:081-3312345

E-mail address: s.suchai.s@hotmail.com

- Are you have received human ethics training?

❑ No ☑ Yes (Certificate as attached)

- Are you have received training for Good Clinical Practice: GCP

  ☐ No ☑ Yes (Certificate as attached)

2) Miss Chulalak Wong-on, PT

☑ Position: Physical Therapist

☑ Department: Rehabilitation Center, Bangkok Hospital Headquarters

☑ Education: Ph.D.

Contact: Rehabilitation Center, Bangkok Hospital Headquarters / 02-3103139

Mobile Phone: 091-801-8181

E-mail address: chulalak_a@hotmail.com

- Are you have received human ethics training?

  ☐ No ☑ Yes (Certificate as attached)

- Are you have received training for Good Clinical Practice: GCP

  ☐ No ☑ Yes (Certificate as attached)

3) Mr. Thanawat Kootanavanichpong, PT

☑ Position: Physical Therapist

☑ Department: Rehabilitation Center, Bangkok Hospital Headquarters

☑ Education: bachelor's degree

Contact: Rehabilitation Center, Bangkok Hospital Headquarters / 02-3103139

Mobile Phone: 081-554-1365

E-mail address: sutheere@gmail.com

- Are you have received human ethics training?

  ☑ No ☐ Yes

- Are you have received training for Good Clinical Practice: GCP

  ☐ No ☑ Yes (Certificate as attached)

4) Mr. Woottichai Utthapong, PT

☑ Position: Deputy head of the Department of Rehabilitation Center

☑ Department: Rehabilitation Center, Bangkok Hospital Headquarters

☑ Education: Ph.D.

Contact: Rehabilitation Center, Bangkok Hospital Headquarters / 02-3103139

Mobile Phone: 089-888-6576

E-mail address: ootthapong@yahoo.com

- Are you have received human ethics training?

  ☐ No ☑ Yes (Certificate as attached)

- Are you have received training for Good Clinical Practice: GCP

  ☐ No ☐ Yes

5) Mr. Daorung Sansuwandee, PT

☑ Position: Physical Therapist

☑ Department: Rehabilitation Center, Bangkok Hospital Headquarters

☑ Education: bachelor's degree

Contact: Rehabilitation Center, Bangkok Hospital Headquarters / 02-3103139

Mobile Phone: 093-354-9990

E-mail address: ptdao.sa@gmail.com

- Are you have received human ethics training?

  ☑ No ☐ Yes

- Are you have received training for Good Clinical Practice: GCP

  ☐ No ☑ Yes (Certificate as attached)

6) Mr. Warut Chaiwong

☑ Position: Biostatistician

☑ Department: Bangkok Research Center

☑ Education: Master's degree in Public Health (Biostatistics)

Contact: Bangkok Research Center, Bangkok Hospital Headquarters

Mobile Phone: 097-319-9592

E-mail address: warut.ch@bdms.co.th

- Are you have received human ethics training?

❑ No ☑ Yes (Certificate as attached)

- Are you have received training for Good Clinical Practice: GCP

❑ No ☑ Yes (Certificate as attached)

7) Chiyanut Surapichpong, MD

☑ Position: general practitioner, GP

☑ Department: Samutprakarn Hospital, Samutprakarn Thailand

❑ Education: Doctor of Medicine (M.D.)

Contact: Samutprakarn Hospital

Mobile Phone: 083-2560777

E-mail address: chaiyanut@hotmail.com

- Are you have received human ethics training?

❑ No ☐ Yes

- Are you have received training for Good Clinical Practice: GCP

❑ No ☑ Yes (Certificate as attached)

3.1 The stakeholder's funding sources/research drugs/research tools.

stakeholder	Co-Investigator
Do you or your family hold >5% shares, work with the company, or earn >300,000 baht/year from funding before the research? (Bangkok Hospital Headquarters and Bangkok Hospital Group salaries excluded)	❑Yes, specify the name.. .... ☑ No, specify the name...................................... ....
Are you in a management or academic position at a company/research funding agency? (Bangkok Hospital Headquarters and the Bangkok Hospital Group excluded)	❑Yes, specify the name.. .... ☑ No, specify the name...................................... ....
Are you an academic, financial, legal advisor, or regular speaker on funding products	❑Yes, specify the name.. .... ☑ No, specify the name...................................... ....
You are involved in the production, purchase, sale, lease, registration, or supply of research-related products.	❑Yes, specify the name.. .... ☑ No, specify the name...................................... ....
Have you received domestic and international conference funding in the previous year or earned speaker fees >300,000 baht? (Bangkok Hospital Headquarters and Bangkok Hospital Group salaries excluded)	❑Yes, specify the name.. .... ☑ No, specify the name...................................... ....

4. Research funding

☑ No research found ❑ On-process for research founding.................. (Specify)

❑ Yes, Research found*

❑ Research funding from the government, specify the source of funding……..

❑ Research funding from the private sector Specifies funding source............

❑Other, Specify…………………………………………..……………………

research funding source

Address…………………..... Research funder's coordinator name..

Tel: ....

E-mail address: ....

5. Research Setting

☑ Single center: Rehabilitation Center, Bangkok Hospital Headquarters

❑ Multiple centers

❑ Specific of Thailand; ………………………………………………………….………………

❑ Collaborate with foreign……………………………………………………………………

6. Project Duration All Process 6 Months

Data Collection 4 Months

7. This project is part of the study or certificate.

☑ No

❑ Yes, Specify…………………………………………

Passed for research proposal examination from Thesis Committee

❑ passed on.....................................

❑ Not yet

8. Full protocol/Proposal

8.1 Background/Rationale

The cardiovascular endurance test evaluates the maximum oxygen consumption of people who walk, climb stairs, run, or do aerobic exercise, which requires the cardiovascular endurance system to transport oxygen to the muscles. (1) The maximum oxygen consumption (VO2max) predicts cardiovascular endurance. (2) and the maximum oxygen intake does not change with exertion. VO2 max is expressed as L/min and mL/kg/min as relative VO2max. VO2max can be estimated using maximal or submaximal tests, direct or indirect methods (3)

The cardiovascular endurance test evaluates Vo2max, which can be measured in the laboratory using a treadmill or bicycle ergometer to assess peak oxygen uptake. It measures the heart rate(HR) at 80-90% of the maximum HR and increases intensity until the maximum level is reached or an adverse event occurs. (4) Besides laboratory testing, the step tests, the 6-minute walk test, and the 2-minute step test are also used in clinical practice. The step test measures peak oxygen consumption. Many test measures aerobic fitness, including the Harvard step test, a "predictive test of VO2max" that starts with participants ascending and descending a platform of 20 inches high for men and 18 inches for women at a rate of 30 completed steps per minute for 5 minutes or until exhaustion. Exhaustion occurs when the participants cannot continuously maintain the stepping rate for 15 seconds. (2,5) Young Men's Christian Association (YMCA) 3-minute step test; This test measures submaximal cardio-respiratory or endurance fitness using a 12 inches (30 cm) step or platform and a metronome. This test begins with alternating stepping at 96steps per minute (4 clicks = one step cycle) for a stepping rate of 24 steps per minute. The participant ascends and descends the platform at the given rate for 3 minutes. (6)

Additionally, the walk test measures submaximal cardio-respiratory or endurance fitness. It was initially developed for patients with lung disease. The original 12-minute walk test (MWT) was reduced to 6 minutes. Evidence indicates that the 6-MWT is better for assessing patients with lung disease exercise response than the 12-MWT. A recent study showed that walking is a more sensitive exercise modality for bronchodilator response in patients with lung disease. (7) The 6-MWT measures a patient's 6-minute walk on a flat surface. It evaluates the global and integrated responses of all

exercise systems, including the pulmonary, cardiovascular, systemic, and peripheral circulations, blood, neuromuscular units, and muscle metabolism. Furthermore, the 6-MWT can be used to evaluate prognostic factors and serially evaluate patient's condition and therapeutic response.

In conclusion, the 6-MWT predicts peak VO2 and functional capacity moderately well in patients with CHF walking <490 m. (8,9) The American Thoracic Society recommended the 6-MWT for assessing functional status and predicting morbidity and mortality rates in patients with heart and lung disease and older adults. (9)

The 2-minute step test (2MST) was initially developed to assess aerobic endurance and functional fitness in the geriatric population. This test is also used for patients with cancer, Alzheimer’s disease, cardiovascular disease (heart failure, hypertension), stroke, renal disease, pulmonary disorder, healthy adults, and mixed populations (fallers, fragile, sarcopenic, and those with cognitive impairment). The literature review found a significant correlation between 2MST and 6-MWT and the 2MST’s ability to specifically evaluate cardiovascular endurance. (5) The therapist instructed the participants to stand next to a wall, mark the heights of their iliac crest and patella, and place a measuring tape at the midpoint between the two on the wall. They were further instructed to step (not run) in one place, raising each knee to the mark on the wall as many times as possible within the 2-minute period and count the number of times the right knee reaches the marked height. (10)

The literature review shows that the cardiovascular endurance standard test is a laboratory test. The high cost of treadmills and bicycles, the need for a specific location, and the need for an expert to administer the test limit its usage for assessing cardiovascular endurance. (4) The Harvard step test has a step height limitation and is inappropriate for older adults. This frequently results in fatigue before the end of the test. (5) The 6-MWT is a standardized cardiovascular endurance test. However, it requires 30-meter corridors, which force patients to turn more frequently, slowing the walking pace and limiting walking distance. Walking for extended periods can cause muscle fatigue, especially in older adults. (11)

The 2-MST accurately assesses cardiopulmonary endurance in patients with heart and lung disease and older adults (12). The American Physical therapy association recommends evaluation of

cardiovascular endurance in patients with COVID-19. (13) A literature review reported that the step test significantly correlates with 6-MWT for evaluating desaturation in patients with heart and lung disease (14)

Additionally, the American Physical Therapy Association recommends the 2-MST for assessing cardiovascular endurance in patients with COVID-19. (13) However, we find it difficult to determine the leg lift's height in clinical practice independently. We developed a new cardiovascular endurance assessment using the 2 -minute marching test (2-MMT) to overcome this limitation. The YMCA step test establishes a leg lift height of 30 cm as rapidly as possible in 2 minutes.

The 2-MMT is a prototype study used to initiate further research in patients with heart and lung problems, including patients with COVID-19 undergoing physical therapy treatment and unable to be assessed in the hospital during COVID-19 pandemic.

Review literature: According to the research proposal attachment

8.2 Objectives

Primary Objective:

To investigate the equivalent test of cardiovascular endurance between a 2-MMT and 6- MWT in healthy volunteers

Secondary Objective

1) To investigate the sensitivity, specificity, and optimal cut-off score of the newly developed cardiovascular endurance test using a 2-MMT
2) To investigate the similarity between a 2-MMT and 6- MWT in terms of exercise induces oxygen desaturation.
3) To investigate between a 2-MMT and 6- MWT in terms of vital sign responsibilities
4) To compare the rate of perceived exertion (RPE) between a 2-MMT and 6- MWT.
5) To compare the level of leg fatigue scale (LFS) between a 2-MMT and 6- MWT.

8.3 Research project type:

☑ Experimental biomedical / Clinical research โปรดระบุ

☑ Experimental procedure/intervention ระบุ; Cardiovascular endurance test in healthy volunteers

❑ High-risk ☑ Minimal risk

8.4 Research design

☑ Randomized-controlled trial (A crossover study)

8.5 Research participants

Sample size calculation

The sample size was estimated using clinical margin = 2 (MCID), (15) with a standard deviation of 8.6, (16) an alpha error probability of 5%, and a statical power of 90%. The results indicated that the required sample size for the Equivalence study is 101 participants for a cross-over design following the formula (17):

$$n = \frac{\left(z_{\alpha} + z_{\frac{\beta}{2}}\right)^2 \sigma_m^2}{2(\delta - |\varepsilon|)^2} = \frac{(1.645 + 1.645)^2 8.6^2}{2(2-0)^2} = 101$$

Note:

δ = Clinical meaningful difference = 2 (MCID)

ε = mean different of VO_{2max} between 2-MST and 6-MWT = 0

σ_m^2 = variance of VO_{2max} = 8.6

z_{α} = Type I error set 5% (alpha = 0.05) = 1.645

$z_{\beta/2}$ = Type II error set 10% (power of test = 90%) = *1.645*

Sample size = 101 sample/sequence

The sample size for 2 sequences = 202 Sample

This includes a dropout rate of 20%; it was with 127 samples/sequence, and both sequences = 254 samples.

Inclusion criteria

Healthy individuals who work at Bangkok Hospital Headquarters were included in the study.

Inclusion criteria include.

1) Women and men between 20 and 50 years
2) Independent activity
3) Height ≥ 150 cm.
4) Normal body mass index ≤ 25 kg/m2
5) Normal chest radiograph in an annual report
6) Normal electrocardiogram in an annual report

Exclusion criteria

1) Abnormal vital signs, including
   - Systolic Blood pressure (SBP) ≥ 140 mmHg
   - Diastolic Blood pressure (DBP) ≥ 90 mmHg
   - HR ≥ 100 bpm
   - Respiratory Rate ≥ 20 bpm
   - Oxygen saturation (SpO2) ≤ 95 %
2) History of COVID-19.
3) History of underlying diseases, including
   (1) Heart disease, cardiovascular disease, myocardial disease, atrial fibrillation, valve disease, congenital heart disease, myocardial infection
   (2) Osteoarthritis
   (3) Chronic obstructive pulmonary disease or Asthma
   (4) Peripheral Neuropathy
   (5) Neuromuscular disease

Withdrawal or termination criteria

Participants can terminate the study or withdraw if they are uncomfortable or change their mind about participating.

participant allocation

All 254 participants were defined as random clusters to avoid contamination bias between conditions A (6-MWT) and B (2-MMT). The clusters were randomized into two arms using a 1:1 ratio.

8.6 Methods

8.6.1 Research Design:

Crossover design: Randomized control trial

8.6.2 Research Methodology

The researcher screened the participants who met the inclusion and exclusion criteria and explained the study's introduction, purpose, methodology, benefits, and complication risk.

Additionally, the researcher inquired about their decision to participate and ensured they signed the consent form.

All data collection and records are as follows.

1) General information includes sex, age, height, weight, underlying, and history of COVID-19.
2) All parameters, including HR, RR, SBP, DBP, SpO2, RPE, and LFS, were recorded 10 min before the test (at 0 min, 5 min, and 10 min)
3) Participants were randomly divided into two conditions using cluster random sampling: Condition A (6-MWT) and Condition B (2-MMT).
4) Participants rested for 10 minutes before the test. The duration of condition A approximate time was 30 minutes, and condition B approximate time was 25 minutes, with 1 day washout period (12). The duration of both tests is approximately 1:40 hours (Figure 1)

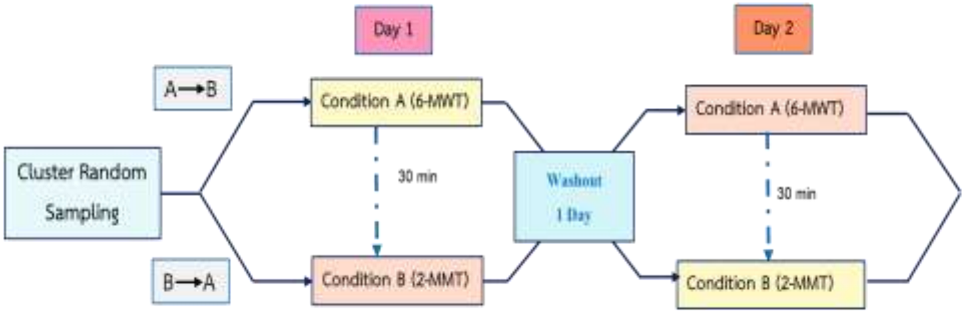

Figure 1: Study design

8.6.3 Study Protocol for LPE

Condition A: The 6-MWT

- **Pre-test**: All parameters, including HR, RR, SBP, DBP, SpO2, RPE, and LFS, were recorded by researchers at 0 minutes, 5 minutes, and 10 minutes before the test
- **During the test**: The 6-MWT was performed indoors on a flat surface in a straight corridor of 30 m, with 180-degree turns every 30 m according to the standard protocol. (9) The cardiopulmonary physical therapist monitored the walk test on stable vital signs and SpO2 >95%. In case of adverse events, the participant must be stopped immediately. The symptoms are; chest pain, RPE > 10, staggering, leg cramps, headache, and sweating. (9)
- **Post-test:** All parameters, including HR, RR, SBP, DBP, SpO2, RPE, and LFS, were recorded by researchers immediately after the test, 5 minutes, and 10 minutes of post-test and the walk distance, for the cardiovascular endurance test, we are assessed by oxygen consumption (VO2Max) and estimated by formula as follows;

VO_{2max} = 70.161 + (0.023 × 6-MWT [m]) - (0.276 × weight [kg]) - (6.79 × sex, where m = 0, f = 1) - (0.193 × resting HR [beats per minute]) - (0.191 × age [y]). (2)

Condition B: 2-MMT

- **Pre-test:** All parameters, including HR, RR, SBP, DBP, SpO2, RPE, and LFS, were recorded by the researchers at 0 minutes, 5 minutes, and 10 minutes of pre-test
- **During the test:** The 2 MMT was developed to count steps in 2 minutes. After the "start" command, a participant marched in position with his knees 30 cm high. The participants had 2 minutes to achieve 30 cm height with as many steps as possible. The participants were allowed to perform a few training steps to verify their ability to complete the task. The participants marched at their own pace; they could slow down or even stop if necessary and continue marching until the 2-minute test time ended. The investigator counted their steps, informed the patients of their trial's duration, and motivated them to do well. The test result was the number of steps with the right foot that touched the ground.

  Participants might slow down or rest if they had severe exercise intolerance (e.g., severe dyspnea, fatigue, or other alarming symptoms) in both tests. However, they were encouraged to resume the test immediately. Adverse events were monitored during and after finishing the test. Both tests were canceled and considered incomplete If chest pain, intolerable dyspnea, leg cramps, staggering, diaphoresis, and ashen appearance occurred.
- **Post-test:** Researchers recorded HR, RR, SBP, DBP, SpO2, RPE, and LFS immediately after the test, 5 minutes, and 10 minutes of post-test and total up and down steps, for the cardiovascular endurance test, VO2Max was estimated by the formula below.

VO2max = 13.341 + 0.138 × total up and down steps

(UDS) – (0.183 × BMI) (18)

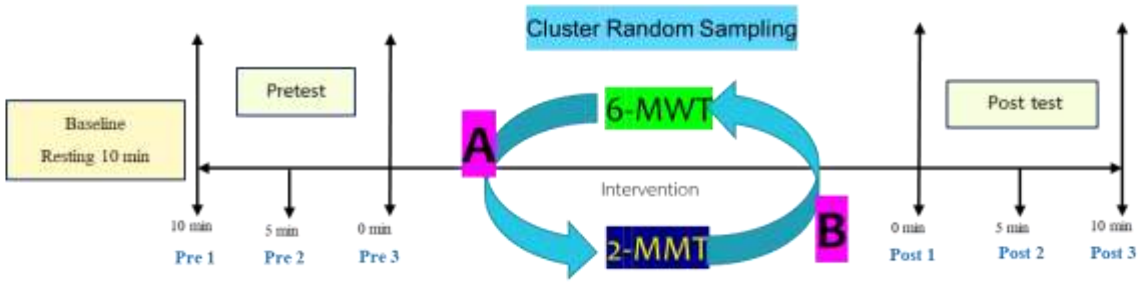

Figure 2: Study protocol

All parameters investigated at pre-test (three times; 0 minutes, 5 minutes, and 10 minutes) and post-test (three times; 0 minutes, 5 minutes, and 10 minutes) were recorded, including;

1) HR
2) RR
3) SBP
4) DBP
5) SpO2
6) Rate of Exertion (RPE) assessed by Borg's CR-10 scale.
7) LFS (modification of Borg's CR-10 scale (12))

8.7 Data collection process

The researcher processed the data collection as follows.

1) Submission of the proposal to the Human Ethics Committee of Bangkok Hospital Headquarters.
2) Data collection according to research methodology in 254 participants and record of all data on case record form.
3) Data analysis according to the research objective and hypothesis.

Data collection must begin after IRB approval.

The researcher uses code designation to identify the participant as participant *ID: starting from 001* - *254*.

8.8 Outcome measurement

The outcome measurement in this study are as follows

1. Cardiovascular endurance assesses by the maximum oxygen consumption (VO_{2max})
   1) The 6–MWT estimated VO2max by the formula (2)

      VO_{2max} = 70.161 + (0.023 × 6-MWT [m]) - (0.276 × weight [kg]) - (6.79 × sex, where m = 0, f = 1) - (0.193 × resting HR [beats per minute]) - (0.191 × age [y]). (2)
   2) The 2 - minute marching test estimated VO2max by formula

      VO_{2max} = 13.341 + 0.138 × total up and down steps (UDS) – (0.183 × BMI) (18)

2. SpO2

   Oxygen desaturation can be identified from the difference in SpO2 at resting and during peak exertion, and we used an oximeter to identify SpO2.

3. Vital sign responsibility

   Vital Sign parameters, including HR, RR, SBP, and DBP, were assessed by Philip Patient Monitor Efficia CM100.
4. RPE

   Borg's CR-10 scale identified the RPE
5. LFS

   The modified Borg's CR-10 scale identified the LFS (12)

8.9 Data analysis

1) Descriptive statistics were used to describe the characteristics of participants, which were presented in mean and standard deviation (mean± standard deviation)
2) The maximum oxygen consumption (VO2max) comparison was analyzed with equivalent test statistics for a 2x2 Cross-Over study.
3) To comparison of oxygen desaturation index between 6-MMT and 2MMT, was analyzed with equivalent test statistics for a 2x2 Cross-Over study.
4) The comparison of vital signs, including HR, RR, SBP, and DBP, was analyzed with equivalent test statistics for a 2x2 Cross-Over study.
5) The RPE was analyzed with equivalent test statistics for a 2x2 Cross-Over study.
6) The LFS was analyzed with equivalent test statistics for a 2x2 Cross-Over study.

8.10 References

1. Chu P, Gotink RA, Yeh GY, Goldie SJ, Hunink MM. The effectiveness of yoga in modifying risk factors for cardiovascular disease and metabolic syndrome: A systematic review and meta-analysis of randomized controlled trials. European Journal of Preventive Cardiology 2016; 23(3): 291-307.
2. Khatkhamwong C, Chidnok W, Jones C. A new cardiovascular fitness test using incremental spot marching exercise in healthy male subjects: Physiological responses. Journal of Medical Technology and Physical Therapy 2019; 31(3): 253-261.
3. Nabi T, Rafiq N, Qayoom O. Assessment of cardiovascular fitness [VO2 max] among medical students by Queens College step test. International Journal of Biomedical and Advance Research 2015; 6(5): 418-421.
4. American College of Sports Medicine. ACSM's guidelines for exercise testing and prescription. Philadelphia: Wolters Kluwer; 2018.
5. Haas F, Sweeney G, Pierre A, Plusch T, Whiteson J. Validation of a 2 minute step test for assessing functional improvement. Open Journal of Therapy and Rehabilitation. 2017; 5(2): 71-81.

6. Kieu NTV, Jung S-J, Shin S-W, Jung H-W, Jung E-S, Won YH, et al. The Validity of the YMCA 3-minute step test for estimating maximal oxygen uptake in healthy Korean and Vietnamese adults. Journal of Lifestyle Medicine 2020; 10(1): 21-29.
7. Cazzola M, Biscione GL, Pasqua F, Crigna G, Appodia M, Cardaci V, et al. Use of 6-min and 12-min walking test for assessing the efficacy of formoterol in COPD. Respiratory Medicine. 2008; 102(10): 1425-1430.
8. Pollentier B, Irons SL, Benedetto CM, DiBenedetto AM, Loton D, Seyler RD, et al. Examination of the six-minute walk test to determine functional capacity in people with chronic heart failure: A systematic review. Cardiopulmonary Physical Therapy Journal 2010; 21: 13-21.
9. ATS statement: guidelines for the six-minute walk test. American Journal of Respiratory and Critical Care Medicine 2002; 166(1): 111-117.
10. Bohannon RW, Crouch RH. Two- minute step test of exercise capacity: Systematic review of procedures, performance, and clinimetric properties. Journal of Geriatric Physical Therapy 2019; 42(2): 105-112.
11. Sirthawong A, Poncumhak P, Sungkamanee S, Manoy P, Boonla O. The optimal cut-off score of the 2-minute step test for prediction of cardiopulmonary endurance in older adults with hypertension. Srinagarind Medical Journal 2019; 34(2): 161-168.
12. Haas F, Sweeney G, Pierre A, Plusch T, Whiteson J. Validation of a 2 minute step test for assessing functional improvement. Open Journal of Therapy and Rehabilitation 2017; 5(2): 71-81.
13. American Physical Therapy Association. (2020). COVID-19 core outcome measures [Internet]. [cited 2021 Nov 20]. Available from: https://webcache.googleusercontent.com/search?q=cache:aoHouIb3uFoJ:https://www.apta.org/contentassets/1a6e0ee7cd25403888d2959c1c8476cd/covid-19-core-outcome-consensus-statement-october-2020.pdf+&cd=12&hl=th&ct=clnk&gl=th

14. Vilarinho R, Caneiras C, Montes AM. Measurement properties of step tests for exercise capacity in COPD: A systematic review. Clinical Rehabilitation 2021; 35(4): 578-588.
15. Chaves Khatkhamwong , Weerapong Chidnok, ChuleeJones. A new cardiovascular fitness test using incremental spot marching exercise in healthy male subjects: Physiological responses. Journal of Medical Technology and Physical Therapy 2019; 31(3): 253-261.
16. Kothmann E, Batterham AM, Owen SJ, Turley AJ, Cheesman M, Parry A, et al. Effect of short-term exercise training on aerobic fitness in patients with abdominal aortic aneurysms: a pilot study. British Journal of Anaesthesia 2009; 103(4): 505-510.
17. Tongprasert S, Wattanapan P. Aerobic capacity of fifth-year medical students at Chiang Mai University. Journal of The Medical Association of Thailand 2007; 90(7): 1411.
18. Chow SC, Shao J, Wang H. Sample size calculation in clinical research. New York: Marcel Dekker, 2003: 67-68.
19. Ricci PA, Cabiddu R, Jürgensen SP, André LD, Oliveira CR, Thommazo-Luporini D, et al. Validation of the two-minute step test in obese with comorbidities and morbidly obese patients. Brazilian Journal of Medical and Biological Research 2019; 52: e8402.

Ethical consideration

9. Participants' characteristics

9.1 Participants' characteristics ☑ Healthy Voluntrees

❑ Patients excluding vulnerable participants

❑ Other: Retrospective chart review

Or ☑ Vulnerable participants* (*The participants who cannot recognize to make decisions or not having the freedom to make decisions on their own*)

Examples ❑ Young children/minors (<18 years old) **

❑ People with disabilities or cognitive/mental disabilities

❑ Emergency or intensive care unit patients, palliative patients

❑ Chronic patients depend on physicians and caregivers

❑ Pregnancy ❑Student ❑ Prisoner

❑Subordinates ❑ People living in foster homes ❑ Illiterate people

❑ Other; ....

9.2 State the reason for being a vulnerable participant (Identification of vulnerability): the vulnerable group may be an employee group or subordinate.

9.3 Justification for using vulnerable participants: Because of subordinates or participants, they are matched with the sample in this study.

9.4 Protection of groups if vulnerable groups are involved: No compulsory participation will affect the annual competency evaluation.

** Who should the researcher ask for consent if a legal representative need one? Please specify..

** for *Children aged* 12-18*, direct consent is required. Parents may use similar informed consent forms with appropriate pronouns accordingly. In addition to obtaining consent from parents or legal representatives (consent), except children with intellectual disabilities, among others.*

*** Children aged 7-12 years may require a direct consent form, known as "acceptance." This document has child-friendly information. Pictures may accompany descriptions. Additionally, parents or legal representatives must consent.*

10. Data use and biological collection of participants/volunteers

10.1 มี Applying for permission to use data or biological products from the repository.

☑ No ❑ Yes

10.2 Data or biological material of participants are requested for future study.

☑ No ❑ Yes

10.3 Specimen sent to outside.

☑ No ❑ Yes

10.4 Received specimen form outside.

☑ No ❑ Yes

11. Recruitment process

11.1 Location: Rehabilitation Center, Bangkok Hospital Headquarters

11.2 Process

11.2.1 A person is responsible for the invitation

☑ Principle Investigator ☑ Co-investigators ☑ Research Assistance

❑ Primary doctors ❑Other: ....

11.2.2 Describe the process of inviting participants in detail, e.g., how to access participants, data access, including the tools for an invitation, and media (if any) with the invitation letter or telephone conversation.

1) The principal investigator or co-investigator invited patients or participated verbally.
2) Used announcements and invitation latter.
3) The principal investigator or co-investigator explains the project background, benefits, and risks of participation.

12. Informed consent process

☑ Have an informed consent process.

- **Information operator:** The principal investigator and co-investigator
- **Information to consent:** The principal investigator and co-investigator
- **Location for consent**: Conference room at rehabilitation center floor 4

  The period for advising participants to consent: 10 minutes for informing them and 5 minutes for consenting.
- **Language:** Thai-language

❑ Execute for Informed consent process

❑ Retrospective chart review

❑ Other: ....

12.1 Informed consent documentation

☑ Participant Information Sheet

☑ Informed Consent Form for whom are > 18 years.

12.2 The process of informed consent from participants / legal representatives

12.2.1. A person is responsible for informed consent.

☑ Principle Investigator ☑ Co-investigators ☑ Research Assistance

❑ Primary doctors ❑Other: ....

12.2.2. Describe the process for informed consent in detail.

1) The principal Investigator or co-investigator explains the project's background, benefits, and risks after reviewing the information sheet and receiving explanations from the researcher.

2) When participants agree to participate, the researcher provides all participants with copies of the information sheet and informed consent forms.

13. Expected Benefits from Research

1) Participants benefit: Participants will know about their cardiovascular system. Both are from the new cardiovascular test, the 2-MMT and 6-MWT

2) Career benefits: This prototype study will be created for future studies in patients with cardiovascular endurance disease and can extend the study results and new knowledge to different medical professional fields.

3) Social benefits: Educational Institutions/academic Institutions can reference and create further research.

14. Risk includes adverse events, effects on participants, and compensation

14.1 Identify adverse event risks and consequences: Risks include physical, psychological, economic, legal, and privacy/confidentiality breaches, including inconvenience and wasted time.

- Physical Risk: fatigue, fainting, or severe symptoms of heart attack
- There may be discomfort If the participant is a subordinate.

14.2 Adverse events prepared for preventive and corrective measures.

- The participant can stop immediately. If they are feeling tired or not ready to participate
- The research team has a certificate of basic life support (BLS) and can administer BLS to participants in case of adverse events. The research will take place in an exercise and cardiac rehabilitation center. which has a safe area and an emergency cart In case of an adverse event

14.3 The person was responsible for expenses/compensation for adverse events from the study. In the case of insurance, please attach a certificate and policy.

- Bangkok Hospital Headquarters was responsible for adverse events.

14.4 The cost of participation and the compensation.

- Because the participants were healthy volunteers from Bangkok Hospital Headquarters, it was a voluntary request with no cost.

14.5 Name of responsibility and contact telephone number in case of adverse events.

1) Sucheela Jisarojito, PM&R Tel: +66 85-811-9338
2) Wuttichai Utthapong, PT. Tel: +66 89-888-6576

14.6 In the case of a clinical trial that uses laboratory tests, pathological results, radiological e results, etc. How does the researcher notify the patient's physician or other physicians who provide treatment?

....

....

14.7 This project has monitored, for example, study monitoring, Data Safety Monitoring Board (DSMB)

❑ Yes: ....

☑ No ❑ N/A

14.8 Other Options for Assessment

☑ Yes: 6 - Minute walk test

❑ No ❑ N/A

14.9 The project plans to analyze terms of project risks.

❑ Yes: ....

❑ No ☑ N/A

15. Related or impacting religion, belief, customs, traditions, or culture. The reputation of the institution, community, locality, or country is researched.

❑ Yes, relevant, specify methods for preventing or mitigating such impacts.

☑ N/A

16. **How to protect the confidentiality of personal information**

16.1 A. Appropriate place, proportional, specific to consent

☑ Yes ❑No...)

16.1 B. Appropriate place, proportional, specific to data collection

☑ Yes ❑No...)

16.2 The method for recording personal data.

❑ No record for personal data

☑ Record personal data.

☑Electronic file ❑Picture ❑VDO

❑Voice record

❑Other...

16.3 If, Record personal data

☑ Save on a computer with a password to protect.

❑ Keep documents/CD/files in a cupboard/drawer with a key lock, and only the researcher has the key to open-close

❑ Destruction of all documents/CDs/files at the end of the research

❑ Return the CD and medical record at the end of the study.

☑ Keep documents/CDs/files for 5 years after the end of the research.

❑ Other..

Identify who has access to the data: principal investigator and co-investigators.

Testimonial of the principal investigator

1. .... The research team and I are named and signed in this document. The research will be conducted as specified in the research project No and approved by the Human Research Ethics Committee, and Informed consent was obtained from all participants. According to ethical principles, the dignity, rights, and welfare of the research participants will be respected.
2. .... If it is necessary to amend the project, we were presented to the Ethics Committee for approval. If the project amendment affects participants, I will be informed of the amendment for all participants.

3. .... I will report adverse events during the study period. According to the regulations of the Ethics Committee within the specified time and will assist in resolving any adverse events during a study period.
4. .... The researcher team and I understand all processes of this study. We can solve problems or adverse events that may occur during the research for the safety and welfare of participants /volunteers.
5. .... When the research is complete, I will summarize the results and notify IRB to complete the project. And suppose the study lasts >1 year. In that case, I will report the progression of the project and apply for a renewal before the expiration.
6. .... The research team and I will not start the research until I have reported the progress and requested a certification renewal before the expiration. The co-investigators understand how to access the data. And will protect the participant's data. Participants can are aware that the research team will keep their information private.

17. Certification from the director of the hospital/director of the center/director of the department or higher that approves the conduct of the research.

Once the Human Research Ethics Committee approves, I certify that the principal investigator, M.D. Suchela Jisarojito has the competence, readiness, and suitability to perform this research.

................Matinee Maipang.............

(Matinee Maipang, MD.)

Deputy Chief Executive Officer (CEO) Group 1

Hospital Director, Bangkok Hospital Headquarters

……22…../…02…/...2022..
